# Palaeoenvironmental drivers of vertebrate community composition in the Belly River Group (Campanian) of Alberta, Canada, with implications for dinosaur biogeography

**DOI:** 10.1186/s12898-016-0106-8

**Published:** 2016-11-15

**Authors:** Thomas M. Cullen, David C. Evans

**Affiliations:** 1 Department of Ecology and Evolutionary Biology, University of Toronto, Toronto, ON Canada; 2 Department of Natural History, Royal Ontario Museum, 100 Queen’s Park, Toronto, ON M5S 2C6 Canada

**Keywords:** Palaeoenvironments, Vertebrate microfossil sites, Palaeoecology, Altitudinal sensitivity, Biogeography, Dinosaurs, Faunal turnover, Latitudinal climate gradients, Belly River Group

## Abstract

**Background:**

The Belly River Group of southern Alberta is one of the best-sampled Late Cretaceous terrestrial faunal assemblages in the world. This system provides a high-resolution biostratigraphic record of terrestrial vertebrate diversity and faunal turnover, and it has considerable potential to be a model system for testing hypotheses of dinosaur palaeoecological dynamics, including important aspects of palaeoecommunity structure, trophic interactions, and responses to environmental change. Vertebrate fossil microsites (assemblages of small bones and teeth concentrated together over a relatively short time and thought to be representative of community composition) offer an unparalleled dataset to better test these hypotheses by ameliorating problems of sample size, geography, and chronostratigraphic control that hamper other palaeoecological analyses. Here, we assembled a comprehensive relative abundance dataset of microsites sampled from the entire Belly River Group and performed a series of analyses to test the influence of environmental factors on site and taxon clustering, and assess the stability of faunal assemblages both temporally and spatially. We also test the long-held idea that populations of large dinosaur taxa were particularly sensitive to small-scale environmental gradients, such as the paralic (coastal) to alluvial (inland) regimes present within the time-equivalent depositional basin of the upper Oldman and lower Dinosaur Park Formations.

**Results:**

Palaeoenvironment (i.e. reconstructed environmental conditions, related to relative amount of alluvial, fluvial, and coastal influence in associated sedimentary strata) was found to be strongly associated with clustering of sites by relative-abundance faunal assemblages, particularly in relation to changes in faunal assemblage composition and marine-terrestrial environmental transitions. Palaeogeography/palaeolandscape were moderately associated to site relative abundance assemblage clustering, with depositional setting and time (i.e. vertical position within stratigraphic unit) more weakly associated. Interestingly, while vertebrate relative abundance assemblages as a whole were strongly correlated with these marine-terrestrial transitions, the dinosaur fauna does not appear to be particularly sensitive to them.

**Conclusions:**

This analysis confirms that depositional setting (i.e. the sediment type/sorting and associated characteristics) has little effect on faunal assemblage composition, in contrast to the effect of changes in the broader palaeoenvironment (e.g. upper vs. lower coastal plain, etc.), with marine-terrestrial transitions driving temporal faunal dynamics within the Belly River Group. The similarity of the dinosaur faunal assemblages between the time-equivalent portions of the Dinosaur Park Formation and Oldman Formation suggests that either these palaeoenvironments are more similar than characterized in the literature, or that the dinosaurs are less sensitive to variation in palaeoenvironment than has often been suggested. A lack of sensitivity to subtle environmental gradients casts doubt on these forces acting as a driver of putative endemism of dinosaur populations in the Late Cretaceous of North America.

**Electronic supplementary material:**

The online version of this article (doi:10.1186/s12898-016-0106-8) contains supplementary material, which is available to authorized users.

## Background

Differences in faunal composition in the Late Cretaceous of Western North America have been hypothesized to reflect adaptation to latitudinal and altitudinal climatic gradients [1–7]. Environmental changes caused by transgression-regression cycles of the Western Interior Sea have been suggested to drive the high diversity and high faunal turnover rates of non-avian dinosaurs [4, 5, 8–11], along with changes in the vertebrate community structure more generally [12–16]. However, global-scale analyses of dinosaurs, as a whole [17] and at the family level [18], indicate that large-scale changes in sea level may not have had a significant influence on broad patterns of diversity, evolution, or migration. This suggests that putative patterns in dinosaur ecology and evolution related to sea level, such as those described from Western North America, may be either the result of other factors, such as sampling biases, or may be occurring on a scale that is too small to be readily detected in such coarse-scale analyses [4]. Previous studies have suggested that the composition and diversity of taxa recovered from specific fossil localities across Western North America varies depending on their distance from the palaeoshoreline of the Western Interior Sea [1–3, 5, 7, 19, 20]. This has led to considerable debate regarding the degree of provinciality/endemism in dinosaur populations [1–7, 9, 10, 14, 15, 19–28], the putatively high diversity and restricted range of dinosaur taxa when compared to modern large mammals [2, 5, 7, 19, 20, 22, 29–37], as well as discussions of niche-partitioning in dinosaurs across environmental gradients in a single depositional basin [2, 5, 10, 19, 20, 23, 24, 29, 30, 38–45]. These noted variations in palaeocommunities over sub-million year timeframes and over relatively small palaeogeographic areas suggests that many taxa, but particularly large-bodied dinosaurs, may have been sensitive to palaeoclimatic and palaeoenvironmental change [1–3, 5, 7, 9, 43, 44, 46]. However, this model has been challenged for reliance on data derived from disjunct geographic areas that are poorly constrained chronostratigraphically [28]. The ability to test hypotheses about dinosaur biogeography, endemism, and environmental sensitivity has historically been difficult, as many species were collected with only limited geological data or stratigraphic information, and were known by very low sample sizes [24, 43, 47], though ongoing work relocating these sites and incorporating them into the broader stratigraphy is ameliorating some of these issues [23, 24, 43, 44, 48].

The Belly River Group of southern Alberta is one of the best-sampled Late Cretaceous vertebrate fossil deposits in the world [44], providing a high-resolution biostratigraphic record of terrestrial vertebrate diversity and faunal turnover, and has considerable potential to be as a model system for testing hypotheses of dinosaur palaeoecological dynamics, including important aspects of palaeoecommunity structure, trophic interactions, and responses to environmental change [48]. The Belly River Group is composed of the Foremost, Oldman, and Dinosaur Park formations, and spans a large portion of the Campanian from a period of time from approximately 79–74 Ma [1, 49, 50]. The full extent of the Belly River Group records two major regional sea level changes in the Western Interior Seaway (the relatively shallow, inland seaway that at its greatest extent stretched from The Arctic Ocean to Gulf of Mexico), the first of which is a regressive event in the Foremost and lower Oldman formations, and the second of which is a major transgressive event recorded in the uppermost Oldman and Dinosaur Park formations that marks the boundary between the Belly River Group and the overlying Bearpaw Formation [49, 50]. The Foremost Formation is the stratigraphically lowest unit within the Belly River Group, and gradationally overlies the marine shales of the Pakowki Formation. The earliest Foremost sediments show considerable marine influence, and the formation, going from lowest to highest outcrops, is generally composed of paralic to non-marine sediments, following a coarsening upwards succession [50, 51]. Conformably overlying the Foremost Formation is the Oldman Formation. The Oldman Formation is broadly considered to represent more fluvial, inland conditions, and is made up of series of upward fining palaeochannel sandstone successions, with a variety of channel top, channel margin, and overbank facies [12, 14, 50, 52]. The amount of exposure of upper Oldman sediments is dependent on sampling location, with southern sites showing a thick succession of strata, and northern sites truncated above the middle Oldman by a regional disconformity [14, 50]. To the north, upper Oldman Formation strata are replaced disconformably by those fo the Dinosaur Park Formation as a result of clastic-wedge replacement [50]. The Dinosaur Park Formation (DPF) is considered to have a greater coastal influence than the underlying Oldman Formation (OM), and is characterized by sandy to muddy, alluvial, estuarine, and paralic facies [12, 49, 50, 52]. The transition to more marine environment in the upper Dinosaur Park Formation is marked by the deposition of the Lethbridge Coal Zone (LCZ), which interfingers with and is overlain by marine shales of the Bearpaw Formation [13, 49, 53]. The pre-LCZ section of the Dinosaur Park Formation in Dinosaur Provincial Park (DPP) is thought to be broadly time-equivalent to the upper Oldman Formation in the Milk River/Manyberries (MRM) regions of southern Alberta [14, 50]. The muddy strata of the upper Oldman Formation in Milk River/Manyberries are typically thought to be more environmentally similar to that of the middle Oldman ‘Comrey sandstone’ (representing a more seasonally arid, inland, fully non-marine fluvial landscape) than to the time-equivalent pre-LCZ Dinosaur Park Formation in DPP (representing a wetter, more marine-influenced coastal plain) [14, 49, 50]. This view has been questioned by other studies suggesting the time-equivalent Oldman and Dinosaur Park formations share a generally wet, coastal environment and that variations in recorded wet-dry signal may represent seasonality and/or spatial variation in local habitat [22, 54]. Interpreting these units is complicated by the existence of heterogeneity in their geographic extent and palaeoenvironment through time, particularly in their relation to the shore of the Western Interior Seaway [5, 8, 19, 20, 24]. Meaningful comparison between these units requires detailed chronostratigraphic control to mitigate the confounding effects of temporal changes in community structure. This is particularly important because the distribution of dinosaurs has been hypothesized to be sensitive to even small-scale palaeoenvironment differences, such as those between the lower and upper coastal plain settings of the time-equivalent Oldman and Dinosaur Park formations [5, 14, 24, 50, 52].

Vertebrate microfossil sites from the Belly River Group have provided a wealth of knowledge on vertebrate palaeoecology [12, 14, 15, 52, 55, 56]. Vertebrate microfossil sites, sensu [57], are useful in overcoming issues of low sample size that commonly hinder palaeontological investigations, as they are both abundant and each site can preserve large numbers of small teeth, bones, and scales of numerous taxa thought to represent much of the vertebrate community composition of a given area [12–16, 53, 55, 58]. These sites are concentrated in a number of ways, with most representing in-channel deposits, crevasse splays, low energy ponds, or shoreface lag deposits [14, 16, 52, 59, 60]. While vertebrate microfossil sites in a given area or formation may represent the same broader palaeoenvironment (e.g. upland fluvial system, lowland coastal plain, etc.), their method of deposition may differ (e.g. flow rates, depositional energy, sediment size/sorting, etc.), and this has led to concerns that microsites with differing depositional setting may not preserve comparable vertebrate material or faunal assemblages [14, 59]. These concerns regarding the effect of depositional setting on the presence/absence and ranked abundances of taxa at a given microvertebrate site have been partially ameliorated by a new taphonomic model for microsite formation suggesting that depositional setting may not play a large role when comparing sites of different type, as channel deposits may represent the short-term erosion and local re-deposition of lower-energy pond deposits [59]. Microsite studies in the Belly River Group of Alberta have so far focused on identifying the taxa present across the region, and assessing any broad trends or associations that may be present through specific stratigraphic intervals [8, 12–14, 16, 53, 58, 61]. These studies have identified aquatic and terrestrial communities, along with some evidence of potential endemism. Additionally, they have demonstrated that terrestrial-marine environmental transitions drive at least some of the changes in vertebrate faunal assemblages, such as an increase in ceratopsians dinosaur abundance correlated with increasing marine influence in the Dinosaur Park Formation of DPP [8], and an inverse relationship between sharks and lissamphibian abundances in the Foremost Formation [16]. However, major quantitative studies have focused on either the transgressive or regressive sequences, and not the entirety of the Belly River Group, resulting in our understanding of the environmental drivers behind microsite faunal assemblage structure remaining incomplete despite the abundance and quality of the available data. In addition, new data suggests that dinosaur species found in the Dinosaur Park Formation of DPP are also found in the time-equivalent sections of the Oldman Formation in MRM [22, 24]. These units have been described as representing different palaeoenvironmental regimes [14, 22, 24, 50]. However, the Oldman Formation is palaeoenvironmentally dynamic throughout its history, shifting from lowland coastal plain to more inland braided rivers, and back to lowland coastal plain [14, 16, 22, 50]. As a result, comparisons of vertebrate microfossil sites can provide an important test of habitat sensitivity in dinosaurs.

Given the ongoing debate regarding the putatively narrow associations of dinosaurs with particular environments, locations, and/or geological formations, this study seeks to use the largest Cretaceous vertebrate microsite dataset yet assembled to first confirm the previously suggested associations between faunal assemblages and differing environments, and then use those as a proxy to test for differences in dinosaur assemblages in the time-equivalent sections of the Dinosaur Park and Oldman formations. We hypothesize that altitudinal (inland vs. coastal) effects will act as the largest driver of faunal assemblage change, following previous results on more limited datasets, with other taphonomic or temporal effects acting minimally on the preserved ecological signal. We also hypothesize that dinosaurs, particularly those of large body size, will not be sensitive to altitudinal change as recorded in the Belly River Group. This hypothesis is based on the resilience to environmental variation and broad latitudinal distributions seen in many groups of large mammals today [2, 7, 32], though it is at odds with much of the literature on dinosaur environmental associations [1–9, 11, 14, 15, 19–24, 26, 27, 42, 43, 62].

## Methods

### Dataset integration and taxonomic consistency

**Fig. 1:**
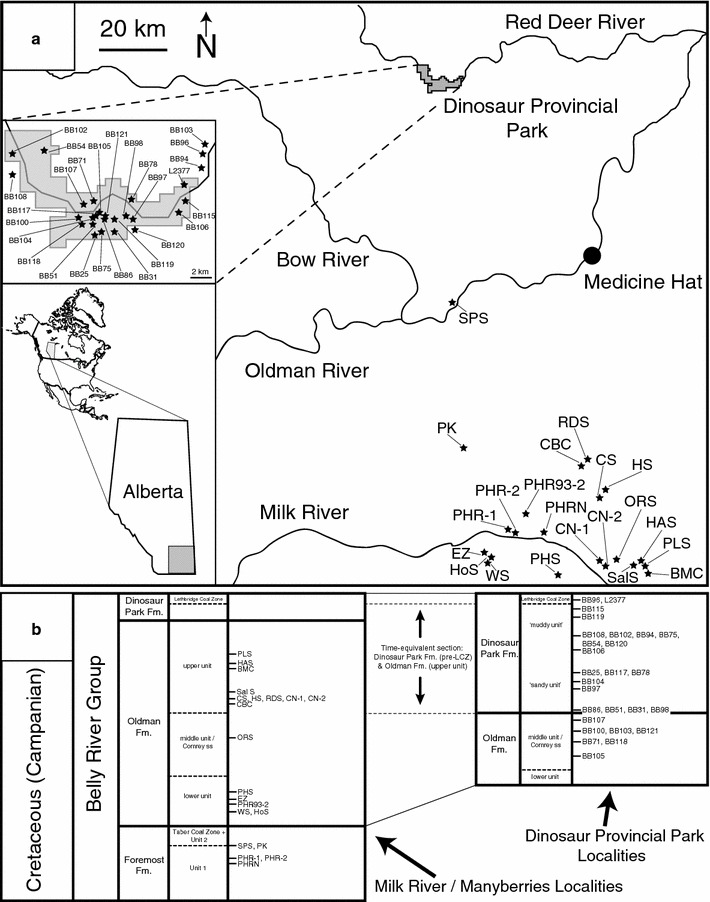
Locality map of Belly River Group sites analyzed in this study. **a** Geographic location of sites in Alberta, in context to regional landmarks; **b** relative stratigraphic positions of sites within the Belly River Group at each sampling region. Map modified from [16] and site locality data compiled from [12, 14, 16]

A dataset (Table 1) of vertebrate microfossil taxon abundance for Belly River Group sites (N = 48) was created through the merger of multiple literature sources [12–14, 16, 53, 58, 61]. These sites span the duration of the Belly River Group (BRG), and were sampled spatially from Dinosaur Provincial Park (DPP) and from the area around the Milk River south of Manyberries (MRM) (Fig. 1). Some revision to the taxonomic categories was required in order to successfully merge these datasets, with specific changes related to differences in the inclusivity of taxonomic categories in each source dataset (e.g. ‘mammals’ vs. genus-level assignment of respective taxa, due to a lack of genus resolution in included DPP data; inclusion of specimens of Allocaudata within Caudata in early studies and separation in later studies, Brinkman pers. comm. 2016), updating terminology to reflect more recent taxonomic revisions (e.g. *Atractosteus* to *Lepisosteus* for BRG gars, ‘teleost D’ to *Coriops*), noting areas of potential future taxonomic revision (e.g. *Myledaphus* to *Myledaphus* + *Pseudomyledaphus*, as the latter may exist in multiple sites under the former name), and revisions to identifications based on discussions with the collectors of the source microsite data (e.g. Pachycephalosaur material being now referred to hypsilophodont, Brinkman pers. comm. 2016) [14, 16, 63, 64].

**Table 1:** Taxon abundance table for each site analyzed in this study, with information derived and standardized from literature [12–14, 16, 53, 58, 61]

^Taxon^	*Myledaphus* *+* *Pseudomyledaphus*	*Protoplatyrhina*	*Hybodus*	*Centrophoroides*	Odontaspididae	*Cretolamna*	*Archaeolamna*	Orectolobidae	*Synechodus*	*Rhinobatos*	*Ischyrhiza*	*Chiloscyllium*	*Squatina*	Elasmobranchii indet.
Site														
BB96 (DPF/LCZ; DPP)	496	79	49	0	5	0	46	3	0	0	12	0	0	2
L2377 (DPF/LCZ; DPP)	1019	356	88	0	91	12	37	38	0	3	16	0	27	4
BB115 (DPF/pre-LCZ; DPP)	243	0	2	0	0	0	0	0	0	0	0	0	0	0
BB119 (DPF/pre-LCZ; DPP)	129	0	0	0	0	0	0	0	0	0	0	0	0	0
BB108 (DPF/pre-LCZ; DPP)	354	0	0	0	0	0	0	0	0	0	0	0	0	0
BB102 (DPF/pre-LCZ; DPP)	948	0	2	0	0	0	0	0	0	0	0	0	0	0
BB94 (DPF/pre-LCZ; DPP)	107	0	0	0	0	0	0	0	0	0	0	0	0	0
BB75 (DPF/pre-LCZ; DPP)	55	0	0	0	0	0	0	0	0	0	0	0	0	0
BB54 (DPF/pre-LCZ; DPP)	218	0	0	0	0	0	0	0	0	0	0	0	0	0
BB120 (DPF/pre-LCZ; DPP)	228	0	0	0	0	0	0	0	0	0	0	0	0	0
BB106 (DPF/pre-LCZ; DPP)	13	0	0	0	0	0	0	0	0	0	0	0	0	0
BB25 (DPF/pre-LCZ; DPP)	54	0	0	0	0	0	0	0	0	0	0	0	0	0
BB117 (DPF/pre-LCZ; DPP)	61	0	0	0	0	0	0	0	0	0	0	0	0	0
BB78 (DPF/pre-LCZ; DPP)	6	0	0	0	0	0	0	0	0	0	0	0	0	0
BB104 (DPF/pre-LCZ; DPP)	110	0	0	0	0	0	0	0	0	0	0	0	0	0
BB97 (DPF/pre-LCZ; DPP)	92	0	0	0	0	0	0	0	0	0	0	0	0	0
BB86 (DPF/pre-LCZ; DPP)	657	0	0	0	0	0	0	0	0	0	0	0	0	0
BB51 (DPF/pre-LCZ; DPP)	163	0	0	0	0	0	0	0	0	0	0	0	0	0
BB31 (DPF/pre-LCZ; DPP)	54	0	0	0	0	0	0	0	0	0	0	0	0	0
BB98 (DPF/pre-LCZ; DPP)	34	0	0	0	0	0	0	0	0	0	0	0	0	0
BB107 (OM/Comrey; DPP)	68	0	0	0	0	0	0	0	0	0	0	0	0	0
BB100 (OM/Comrey; DPP)	93	0	0	0	0	0	0	0	0	0	0	0	0	0
BB103 (OM/Comrey; DPP)	9	0	0	0	0	0	0	0	0	0	0	0	0	0
BB121 (OM/Comrey; DPP)	52	0	0	0	0	0	0	0	0	0	0	0	0	0
BB71 (OM/Comrey; DPP)	9	0	0	0	0	0	0	0	0	0	0	0	0	0
BB118 (OM/Comrey; DPP)	2	0	0	0	0	0	0	0	0	0	0	0	0	0
BB105 (OM/Comrey; DPP)	20	0	0	0	0	0	0	0	0	0	0	0	0	0
PLS (OM/upper; MRM)	14	0	0	0	0	0	0	0	0	0	0	0	0	0
HAS (OM/upper; MRM)	2	0	0	0	0	0	0	0	0	0	0	0	0	0
BMC (OM/upper; MRM)	0	0	0	0	0	0	0	0	0	0	0	0	0	0
SalS (OM/upper; MRM)	1	0	0	0	0	0	0	0	0	0	0	0	0	0
CS (OM/upper; MRM)	2	0	0	0	0	0	0	0	0	0	0	1	0	0
HS (OM/upper; MRM)	6	0	0	0	0	0	0	0	0	0	0	0	0	0
RDS (OM/upper; MRM)	102	0	0	0	0	0	0	0	0	0	0	0	0	0
CN-1 (OM/upper; MRM)	2	0	0	0	0	0	0	0	0	0	0	1	0	0
CN-2 (OM/upper; MRM)	0	0	0	0	0	0	0	0	0	0	0	0	0	0
CBC (OM/upper; MRM)	1	0	0	0	0	0	0	0	0	0	0	0	0	0
ORS (OM/Comrey; MRM)	2	0	0	0	0	0	0	0	0	0	0	0	0	0
PHS (OM/lower; MRM)	19	0	0	0	0	0	0	0	0	0	0	0	0	0
EZ (OM/lower; MRM)	8	0	0	0	0	0	0	0	0	0	0	0	0	0
PHR93-2 (OM/lower; MRM)	24	0	0	0	0	0	0	0	0	0	0	1	0	0
WS (OM/lower; MRM)	9	0	0	0	0	0	0	0	0	0	0	1	0	0
HoS (OM/lower; MRM)	15	0	0	0	0	0	0	0	0	0	0	0	0	0
SPS (Foremost; MRM)	235	0	0	0	0	0	0	0	0	0	0	5	0	0
PK (Foremost; MRM)	29	0	1	0	2	0	3	0	0	0	0	0	0	2
PHR-1 (Foremost; MRM)	1952	0	10	0	23	0	0	0	0	0	8	0	1	0
PHR-2 (Foremost; MRM)	2164	0	15	0	79	0	10	1	1	9	16	6	8	0
PHRN (Foremost; MRM)	3780	382	18	15	50	4	27	5	10	3	20	0	34	690

^Taxon^	*Elasmodus* sp.	‘Holostean A’	‘Holostean B’	Acipenseriformes	*Belonostomus*	*Lepisosteus*	Amiidae	Phyllodontidae	*Paratarpon*	Esocidae	*Enchodus*	*Coriops*	Teleostei	Anura
Site														
BB96 (DPF/LCZ; DPP)	5	0	0	14	0	0	0	215	0	0	4	0	0	0
L2377 (DPF/LCZ; DPP)	40	0	0	1	0	0	0	679	0	0	0	0	3	0
BB115 (DPF/pre-LCZ; DPP)	0	0	3	2	2	39	0	16	1	0	0	1	3	0
BB119 (DPF/pre-LCZ; DPP)	0	26	2	1	2	204	1	6	0	1	0	0	1	1
BB108 (DPF/pre-LCZ; DPP)	0	13	0	0	5	90	1	6	0	5	0	1	4	7
BB102 (DPF/pre-LCZ; DPP)	0	668	11	39	22	2714	18	36	0	14	0	17	56	23
BB94 (DPF/pre-LCZ; DPP)	0	44	1	10	13	161	5	4	0	2	0	2	15	3
BB75 (DPF/pre-LCZ; DPP)	0	116	1	5	10	157	2	4	1	3	0	4	11	2
BB54 (DPF/pre-LCZ; DPP)	0	1582	196	15	5	742	149	13	0	35	0	155	331	8
BB120 (DPF/pre-LCZ; DPP)	0	105	1	8	16	155	9	12	0	6	0	47	32	14
BB106 (DPF/pre-LCZ; DPP)	0	66	0	4	1	91	7	6	0	3	0	8	49	29
BB25 (DPF/pre-LCZ; DPP)	0	80	1	1	6	29	12	1	0	4	0	6	28	13
BB117 (DPF/pre-LCZ; DPP)	0	17	0	1	6	41	3	4	0	4	0	3	19	8
BB78 (DPF/pre-LCZ; DPP)	0	175	0	0	0	130	5	35	0	3	0	33	57	24
BB104 (DPF/pre-LCZ; DPP)	0	213	3	7	3	16	42	22	0	14	0	56	163	112
BB97 (DPF/pre-LCZ; DPP)	0	86	1	0	3	62	7	6	0	8	0	16	40	35
BB86 (DPF/pre-LCZ; DPP)	0	223	6	9	12	25	58	11	0	16	0	65	190	177
BB51 (DPF/pre-LCZ; DPP)	0	218	0	6	8	10	19	4	0	2	0	23	119	20
BB31 (DPF/pre-LCZ; DPP)	0	255	0	1	1	19	11	3	0	12	0	33	79	64
BB98 (DPF/pre-LCZ; DPP)	0	124	0	0	0	3	28	10	0	6	0	25	63	65
BB107 (OM/Comrey; DPP)	0	334	0	3	8	48	18	14	0	10	0	30	136	24
BB100 (OM/Comrey; DPP)	0	268	0	5	2	46	17	11	0	15	0	34	109	77
BB103 (OM/Comrey; DPP)	0	359	0	0	0	379	16	14	0	17	0	73	213	85
BB121 (OM/Comrey; DPP)	0	62	0	9	10	152	9	6	0	2	0	20	60	6
BB71 (OM/Comrey; DPP)	0	36	0	3	1	12	6	3	0	0	0	6	15	5
BB118 (OM/Comrey; DPP)	0	105	0	0	0	4	3	2	0	5	0	7	26	20
BB105 (OM/Comrey; DPP)	0	212	0	0	0	46	11	9	0	16	0	123	51	89
PLS (OM/upper; MRM)	0	9	0	0	0	30	3	2	0	5	0	0	1	15
HAS (OM/upper; MRM)	0	32	0	0	0	126	17	8	0	10	0	41	141	92
BMC (OM/upper; MRM)	0	0	0	0	0	6	0	0	0	0	0	2	10	35
SalS (OM/upper; MRM)	0	32	0	0	0	10	44	0	0	7	0	20	48	40
CS (OM/upper; MRM)	0	49	0	0	0	169	0	4	0	18	0	18	6	66
HS (OM/upper; MRM)	0	36	0	0	0	107	2	9	0	13	0	22	197	90
RDS (OM/upper; MRM)	0	73	0	0	0	0	1	9	0	15	0	16	34	44
CN-1 (OM/upper; MRM)	0	57	0	0	0	30	0	16	0	22	0	48	230	102
CN-2 (OM/upper; MRM)	0	5	0	0	0	14	0	0	0	2	0	8	36	33
CBC (OM/upper; MRM)	0	19	0	0	6	123	7	12	0	10	0	32	73	147
ORS (OM/Comrey; MRM)	0	55	0	0	0	10	0	2	0	10	0	13	144	33
PHS (OM/lower; MRM)	0	1	0	6	0	339	0	45	0	1	0	35	148	29
EZ (OM/lower; MRM)	0	1	4	0	0	147	18	8	0	4	0	42	150	43
PHR93-2 (OM/lower; MRM)	0	31	5	4	0	126	14	19	0	6	0	43	101	70
WS (OM/lower; MRM)	0	138	25	0	11	404	11	16	0	15	0	63	225	75
HoS (OM/lower; MRM)	0	54	0	6	0	1001	10	12	0	7	0	67	100	47
SPS (Foremost; MRM)	0	229	45	14	0	204	5	139	0	29	0	22	46	24
PK (Foremost; MRM)	4	0	6	0	0	43	0	0	0	0	0	0	1	1
PHR-1 (Foremost; MRM)	0	94	355	90	185	2463	9	1156	0	4	0	10	89	33
PHR-2 (Foremost; MRM)	0	22	166	25	160	834	17	2016	0	7	0	30	265	31
PHRN (Foremost; MRM)	0	8	56	2	0	143	0	2281	0	0	0	0	305	10

^Taxon^	Caudata + Allocaudata	Mosasauridae	Squamata	Plesiosauria	Testudines indet.	Solemydidae	*Basilemys*	Trionychidae	*Adocus*	Chelydridae	Baenidae	*Champsosaurus*	Eusuchia	Ceratopsidae
Site														
BB96 (DPF/LCZ; DPP)	0	1	0	2	0	0	0	0	0	0	0	0	1	1
L2377 (DPF/LCZ; DPP)	0	0	0	1	0	0	0	0	0	0	0	1	1	0
BB115 (DPF/pre-LCZ; DPP)	13	0	3	0	0	0	1	8	0	0	3	14	15	0
BB119 (DPF/pre-LCZ; DPP)	13	0	0	0	0	0	0	15	0	1	1	15	29	8
BB108 (DPF/pre-LCZ; DPP)	25	0	11	0	0	0	2	10	0	3	4	7	96	6
BB102 (DPF/pre-LCZ; DPP)	244	0	69	0	0	0	2	79	0	13	47	93	414	37
BB94 (DPF/pre-LCZ; DPP)	45	0	7	0	0	0	0	25	0	3	16	7	27	7
BB75 (DPF/pre-LCZ; DPP)	47	0	11	0	0	0	2	15	0	2	5	15	28	4
BB54 (DPF/pre-LCZ; DPP)	347	0	12	0	0	0	0	12	0	5	6	109	53	8
BB120 (DPF/pre-LCZ; DPP)	283	0	17	0	0	0	0	9	0	4	10	41	36	6
BB106 (DPF/pre-LCZ; DPP)	125	0	9	0	0	0	0	38	0	3	14	14	54	7
BB25 (DPF/pre-LCZ; DPP)	120	0	5	0	0	0	0	4	0	3	0	7	7	11
BB117 (DPF/pre-LCZ; DPP)	64	0	3	0	0	0	0	5	0	0	8	4	24	4
BB78 (DPF/pre-LCZ; DPP)	194	0	22	0	0	0	0	31	0	5	3	25	59	13
BB104 (DPF/pre-LCZ; DPP)	340	0	27	0	0	0	0	10	0	11	12	26	30	21
BB97 (DPF/pre-LCZ; DPP)	266	0	12	0	0	0	0	12	0	4	7	7	25	7
BB86 (DPF/pre-LCZ; DPP)	550	0	61	0	0	0	0	28	0	21	30	91	123	9
BB51 (DPF/pre-LCZ; DPP)	235	0	25	0	0	0	0	1	0	3	5	68	33	0
BB31 (DPF/pre-LCZ; DPP)	178	0	13	0	0	0	0	29	0	1	1	55	13	7
BB98 (DPF/pre-LCZ; DPP)	148	0	5	0	0	0	0	15	0	1	0	24	5	3
BB107 (OM/Comrey; DPP)	166	0	14	0	0	0	0	7	0	0	24	24	21	0
BB100 (OM/Comrey; DPP)	296	0	18	0	0	0	0	12	0	2	4	33	29	9
BB103 (OM/Comrey; DPP)	322	0	46	0	0	0	0	15	0	3	0	16	32	0
BB121 (OM/Comrey; DPP)	93	0	7	0	0	0	0	19	0	0	3	8	61	0
BB71 (OM/Comrey; DPP)	33	0	2	0	0	0	0	5	0	0	4	2	7	1
BB118 (OM/Comrey; DPP)	68	0	5	0	0	0	0	12	0	0	1	6	15	4
BB105 (OM/Comrey; DPP)	592	0	35	0	0	0	0	14	0	12	2	13	31	0
PLS (OM/upper; MRM)	39	0	8	0	0	0	0	3	0	2	2	10	30	6
HAS (OM/upper; MRM)	474	0	9	0	0	0	0	3	0	0	5	6	21	10
BMC (OM/upper; MRM)	59	0	9	0	0	0	0	6	0	12	2	0	1	5
SalS (OM/upper; MRM)	105	0	5	0	0	0	0	1	0	3	4	7	20	15
CS (OM/upper; MRM)	378	0	18	0	0	0	0	18	7	15	16	37	93	10
HS (OM/upper; MRM)	393	0	27	0	0	0	0	14	0	1	6	14	82	12
RDS (OM/upper; MRM)	80	0	7	0	0	0	0	14	0	10	14	9	118	19
CN-1 (OM/upper; MRM)	425	0	11	0	0	0	0	6	0	9	5	1	11	18
CN-2 (OM/upper; MRM)	84	0	8	0	0	0	0	4	0	15	8	2	5	2
CBC (OM/upper; MRM)	158	0	11	0	0	0	0	17	0	6	6	2	21	17
ORS (OM/Comrey; MRM)	284	0	9	0	0	0	0	12	0	23	10	4	4	5
PHS (OM/lower; MRM)	73	0	25	0	0	0	0	24	15	0	13	27	51	2
EZ (OM/lower; MRM)	82	0	60	0	0	0	0	6	4	2	1	6	13	11
PHR93-2 (OM/lower; MRM)	223	0	11	0	0	0	0	2	0	0	4	18	20	10
WS (OM/lower; MRM)	317	0	45	0	0	0	0	14	8	5	11	214	82	8
HoS (OM/lower; MRM)	250	0	8	0	0	0	0	2	13	14	2	17	31	3
SPS (Foremost; MRM)	409	0	7	0	0	0	0	1	0	4	18	50	38	2
PK (Foremost; MRM)	1	1	5	0	3	35	2	24	9	1	8	28	51	2
PHR-1 (Foremost; MRM)	41	0	12	0	0	1	0	106	129	21	93	97	122	52
PHR-2 (Foremost; MRM)	103	0	11	0	0	0	0	7	3	1	2	213	205	12
PHRN (Foremost; MRM)	24	0	0	0	0	272	0	21	15	0	2	64	151	4

^Taxon^	Ankylosauria	Hypsilophodont	Hadrosauridae	Theropoda indet.	Dromaeosauridae	Saurornitholestinae	*Richardoestesia*	*Troodon*	*Paronychodon*	Tyrannosauridae	cf. Aves	Mammalia
Site												
BB96 (DPF/LCZ; DPP)	0	0	0	0	0	0	0	0	0	0	0	0
L2377 (DPF/LCZ; DPP)	0	0	0	0	0	0	0	0	0	0	1	1
BB115 (DPF/pre-LCZ; DPP)	1	0	21	0	1	0	0	0	0	1	0	1
BB119 (DPF/pre-LCZ; DPP)	0	0	56	0	0	2	0	0	0	2	0	0
BB108 (DPF/pre-LCZ; DPP)	38	0	110	0	0	35	0	2	0	4	0	3
BB102 (DPF/pre-LCZ; DPP)	126	0	276	0	1	47	0	3	0	2	0	36
BB94 (DPF/pre-LCZ; DPP)	3	0	93	0	1	7	0	0	0	1	0	11
BB75 (DPF/pre-LCZ; DPP)	3	0	81	0	1	0	0	0	0	1	0	10
BB54 (DPF/pre-LCZ; DPP)	1	0	13	0	1	8	0	0	0	0	0	8
BB120 (DPF/pre-LCZ; DPP)	3	0	113	0	0	1	0	0	0	3	0	24
BB106 (DPF/pre-LCZ; DPP)	25	0	100	0	2	8	0	1	0	1	0	1
BB25 (DPF/pre-LCZ; DPP)	6	0	73	0	1	4	0	0	0	3	0	3
BB117 (DPF/pre-LCZ; DPP)	13	0	67	0	0	6	0	0	0	1	0	2
BB78 (DPF/pre-LCZ; DPP)	8	0	174	0	1	8	0	1	0	1	0	0
BB104 (DPF/pre-LCZ; DPP)	14	0	186	0	1	17	0	1	0	1	0	15
BB97 (DPF/pre-LCZ; DPP)	5	2	121	0	1	7	0	0	0	3	0	2
BB86 (DPF/pre-LCZ; DPP)	76	4	424	0	2	43	0	4	0	1	0	9
BB51 (DPF/pre-LCZ; DPP)	12	3	116	0	2	9	0	0	0	0	0	7
BB31 (DPF/pre-LCZ; DPP)	11	3	104	0	1	21	0	0	0	5	0	8
BB98 (DPF/pre-LCZ; DPP)	6	0	106	0	0	8	0	0	0	1	0	7
BB107 (OM/Comrey; DPP)	75	1	65	0	0	1	0	1	0	2	0	7
BB100 (OM/Comrey; DPP)	69	1	161	0	0	17	0	0	0	3	0	4
BB103 (OM/Comrey; DPP)	342	2	263	0	3	4	0	3	0	5	0	16
BB121 (OM/Comrey; DPP)	13	0	92	0	0	6	0	1	0	1	0	2
BB71 (OM/Comrey; DPP)	3	0	26	0	0	2	0	2	0	0	0	1
BB118 (OM/Comrey; DPP)	8	0	62	0	0	7	0	1	0	0	0	1
BB105 (OM/Comrey; DPP)	11	4	418	0	1	28	0	3	0	3	0	26
PLS (OM/upper; MRM)	10	1	128	0	0	25	1	2	2	3	1	6
HAS (OM/upper; MRM)	5	0	268	0	1	8	2	0	2	3	2	13
BMC (OM/upper; MRM)	1	3	54	0	0	4	0	0	1	4	0	19
SalS (OM/upper; MRM)	1	0	132	0	0	5	0	0	1	2	0	6
CS (OM/upper; MRM)	5	2	219	0	0	6	2	1	1	4	2	9
HS (OM/upper; MRM)	2	1	108	0	0	4	2	0	3	1	4	7
RDS (OM/upper; MRM)	6	2	295	0	2	41	7	12	0	4	2	6
CN-1 (OM/upper; MRM)	0	0	136	0	0	10	0	2	0	3	2	8
CN-2 (OM/upper; MRM)	1	0	50	0	0	1	2	1	1	1	1	5
CBC (OM/upper; MRM)	1	0	87	0	0	7	3	4	0	3	0	12
ORS (OM/Comrey; MRM)	5	0	99	0	1	1	2	0	1	1	3	8
PHS (OM/lower; MRM)	2	0	93	0	0	7	0	0	1	2	0	9
EZ (OM/lower; MRM)	1	1	101	0	0	1	0	0	0	1	1	4
PHR93-2 (OM/lower; MRM)	0	0	65	0	0	11	0	0	1	0	1	3
WS (OM/lower; MRM)	0	1	61	0	0	7	0	0	2	3	3	8
HoS (OM/lower; MRM)	2	0	83	0	0	6	2	0	0	2	0	7
SPS (Foremost; MRM)	2	2	162	0	0	2	0	0	2	1	3	38
PK (Foremost; MRM)	6	1	6	0	0	4	0	0	0	1	0	0
PHR-1 (Foremost; MRM)	8	2	178	0	0	7	3	0	0	7	3	11
PHR-2 (Foremost; MRM)	8	0	263	0	0	18	3	0	1	9	0	9
PHRN (Foremost; MRM)	8	0	6	5	0	0	1	0	0	1	1	2

### Dataset standardization and R- vs. Q-mode cluster analysis

The combined data matrix was rarefied in R using functions contained within the ‘vegan’ package [65]. This was done to compare sampling intensity between sites, and found that few sample issues exist between sites, though most sites are likely somewhat undersampled compared to their theoretical optimum. Rarefied data were further Wisconsin double standardized after conversion to relative abundances, again through use of the functions contained in the ‘vegan’ package [65]. The standardized relative abundance dataset was converted to the percentage-difference dissimilarity index (also referred to as Bray-Curtis) [66]. R- vs. Q-mode cluster analyses were generated for the resultant dissimilarity dataset, with UPGMA linkage, using the ‘tabasco’ function of the ‘vegan’ R package [65]. DPP and Milk River sites were identified on the resulting plots, and major site/taxon clusters were highlighted.

The R- vs. Q-mode cluster analysis was repeated for three sub-sample analyses focused on the time-equivalent upper Oldman and Dinosaur Park formations, one including only dinosaur proportions, another including only theropod proportions, and a third including proportions of all non-dinosaurs shared between these sites. The first two subsamples contained different source data (Additional file 1) for Dinosaur Provincial Park sites than the comparisons of total vertebrate assemblages, being derived from the re-sampled dinosaur material of Brinkman et al. [8] instead of Brinkman [12]. The dataset of Brinkman et al. [8] allows for more detailed taxonomic comparisons when restricted to dinosaur data (due to the inclusion of several additional small theropod genera), but is not considered appropriate for the broader vertebrate faunal comparisons due to the lack of methodological consistency (associated with the targeted nature of this additional dinosaur sampling) relative to the faunal data for other vertebrate material in Dinosaur Provincial Park sites. The non-dinosaur subsample used the same source data as the primary analysis of all vertebrates, the only difference being the removal of dinosaurs from the dataset. This last analysis was performed to confirm the effect of dinosaurs on overall trends in the data, and avoid possible issues of circularity in interpreting the dinosaur data in isolation.

### Environmental factors and redundancy analysis

Data relating to the palaeoenvironmental setting (marine, transitional, terrestrial—paralic/lower coastal plain, terrestrial—alluvial/upper coastal plain), palaeogeography (Dinosaur Provincial Park localities, Milk River localities), stratigraphic interval (Foremost Formation, lower Oldman Formation, middle Oldman Formation or ‘Comrey sandstone’, time-equivalent upper Oldman and pre-LCZ Dinosaur Park formations, Lethbridge Coal Zone of Dinosaur Park Formation), and depositional setting (shoreface deposit, crevasse splay deposit, in-channel deposit) were taken from the literature [12–14, 16, 52, 53, 58, 61] and assembled into an environmental data matrix (Additional file 2). Though the palaeogeographical separation between the sampling localities is relatively small in the context of latitudinal climate gradients (~150 km apart, with Dinosaur Provincial Park at ~50.75° latitude and Milk River at ~49.15° latitude), it is of a similar magnitude to previous analyses of vertebrate endemism in microsites [8, 14–16, 53, 58] and endemism/provinciality of large dinosaur macrofossils [1, 5, 19, 20, 37, 43, 44]. The environmental matrix and the dissimilarity matrix generated for the cluster analyses were then ordinated via redundancy analysis (RDA) in order to assess the relationship between each environmental variable and the clustering of site faunal assemblages.

### Pair-wise site assemblage similarity

Relative abundance data of site faunal assemblages were split into two smaller datasets corresponding to sampling location (DPP vs. MRM), and ordered stratigraphically. The proportions of each taxonomic group were then plotted and compared using the R packages ‘ggplot2’ and ‘reshape2’ [67, 68]. Additionally, pair-wise Bray-Curtis similarity values were computed for sites from each sampling area using the ‘fossil’ package [69], and plotted as curves showing relative changes in site similarity through stratigraphy. Average faunal proportions for each stratigraphic interval were also produced and plotted.

This was repeated in three sub-sampling analyses focused on the time-equivalent upper Oldman and Dinosaur Park formations, one including only dinosaur proportions, another including only theropod proportions, and one including non-dinosaur proportions. As with the R- vs. Q-mode subsamples, the first two sub-sample analyses used the re-sampled dataset of Dinosaur Provincial Park sites (from Brinkman et al. [8]), for the same purpose of more specific dinosaur taxon comparability, while the third sub-sample used the primary dataset (with dinosaurs removed).

## Results

### R- vs. Q-mode cluster analysis

**Fig. 2:**
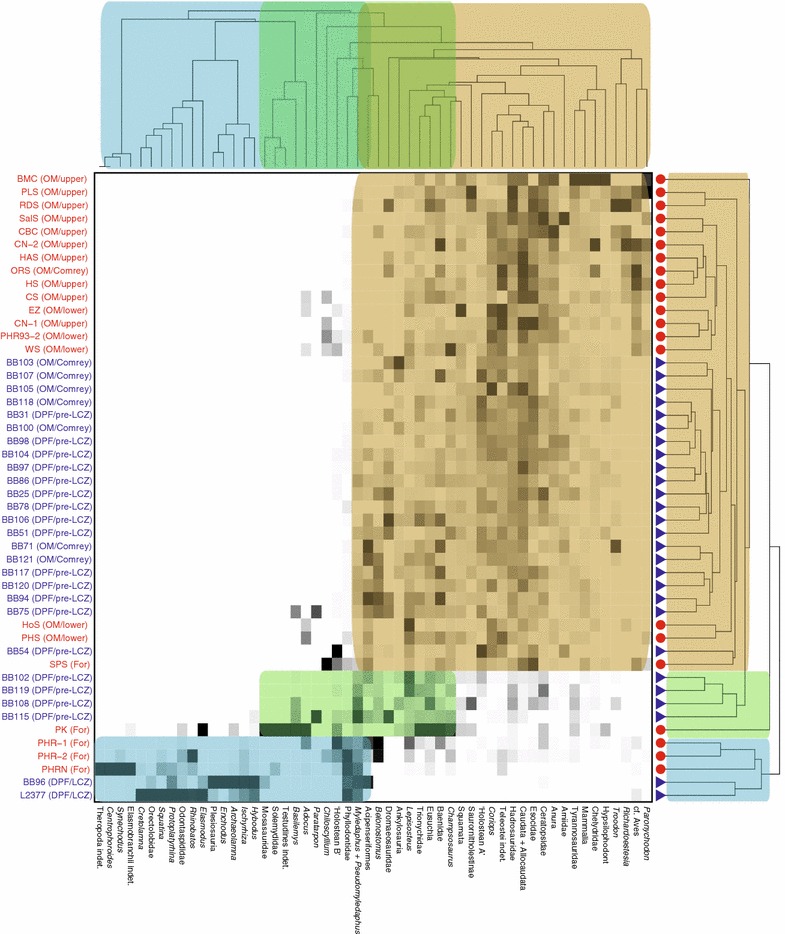
R-mode vs. Q-mode cluster analysis of Belly River vertebrate microfossil sites. Dinosaur Provincial Park sites indicated with *blue text* and *triangles*. Milk River/Manyberries sites indicated with *red text* and *circles*. Coloured regions note environments associated with indicated sites and taxa: *yellow* terrestrial, *green* transitional, *blue* marine

Cluster analyses of sites (Q-mode) and taxa (R-mode) were performed and compared to identify major clustering trends among Belly River Group microsites (Fig. 2). Two primary site clusters were identified, with an additional grade of sites between them. The largest site cluster (yellow highlighted component in Fig. 2) contains all Oldman Formation sites (N = 23), along with a majority of the pre-LCZ Dinosaur Park Formation sites (N = 14, out of a possible 18), and one Foremost Formation site (‘SPS’). This cluster contains two large sub-clusters, which broadly group sites based on their sampling region (either DPP or MRM). The second primary site cluster (blue highlighted component in Fig. 2) contains the three stratigraphically lowest Foremost Formation sites (‘PHR-1’, ‘PHR-2’, ‘PHRN’) and both Lethbridge Coal Zone sites (‘BB96’, ‘L2377’). The grade of sites (green highlighted component) situated between the two primary clusters contains one Foremost Formation site (‘PK’) and four of the stratigraphically highest pre-LCZ Dinosaur Park Formation sites (‘BB102’, ‘BB119’, ‘BB108’, ‘BB115’). These stratigraphically high pre-LCZ Dinosaur Park Formation sites (along with ‘BB94’, ‘BB75’, ‘BB54’, and ‘BB120’, situated in the yellow highlighted component of Fig. 2) are positioned near the locally-variable conformable boundary between the informal lower ‘sandy’ and upper ‘muddy’ units within the pre-LCZ Dinosaur Park Formation, a transition thought to indicate the acceleration of the transgressive sequence leading into the LCZ and Bearpaw Formation [12, 50, 52]. The two primary site clusters correspond broadly to the clustering of taxa in the R-mode analysis, with the larger site cluster associated (yellow in Fig. 2) most strongly with lissamphibians (e.g. Caudata + Allocaudata), dinosaurs (e.g. Hadrosauridae, Ceratopsidae, *Troodon*, Dromaeosauridae, cf. Aves), and actinopterygians (e.g. ‘Holostean A’, *Coriops*, Teleostei, Esocidae), and the smaller primary site cluster (blue in Fig. 2) most strongly associated with batoids (e.g. *Myledaphus* + *Pseudomyledaphus*, *Ischyrhiza*, *Protoplatyrhina, Rhinobatos*), sharks (e.g. *Hybodus*, *Archaeolamna*, *Odontaspidae*), and actinopterygians (e.g. *Belonostomus*, *Enchodus*, Phyllodontidae). The sub-clusters within the larger (yellow in Fig. 2) of the two primary clusters are broadly similar in taxonomic composition, with the only major difference being the lack of several taxa (e.g. *Paronychodon*, *Richardoestesia*, cf. Aves) in Dinosaur Provincial Park sites. The grade of sites (green in Fig. 2) not included in the two primary clusters is associated with aquatic taxa present to varying degrees in both other clusters (e.g. *Myledaphus* + *Pseudomyledaphus*, *Lepisosteus*, Eusuchia, Baenidae, *Champsosaurus*).

### Redundancy analysis

**Fig. 3:**
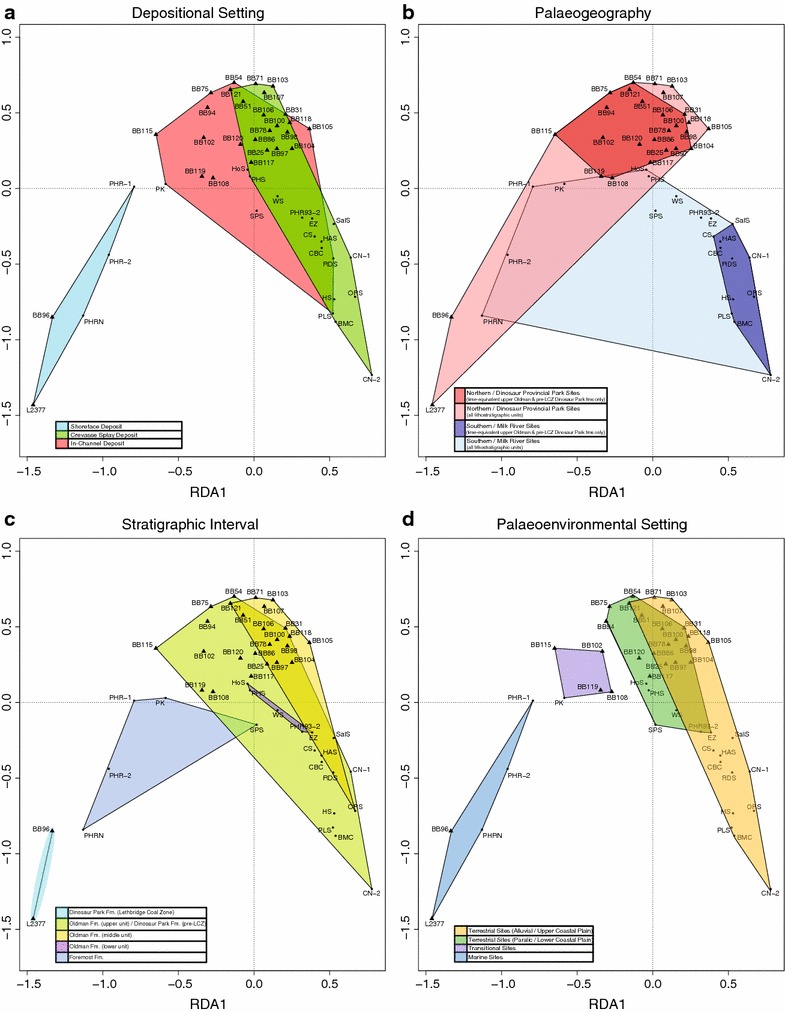
Redundancy analysis of Belly River Group vertebrate microfossil site data and associated environmental factors. **a** Depositional setting; **b** palaeogeographic sampling location; **c** stratigraphic interval; **d** palaeoenvironmental setting. Subdivisions within each environmental factor noted in associated legends

Redundancy analysis (RDA) was carried out on the percent difference dissimilarity matrix computed from the Belly River Group microsite relative abundance dataset, with stratigraphic interval (a proxy for temporal change), depositional setting (site-specific sedimentological characteristics), palaeogeographic sampling location (DPP or MRM), and palaeoenvironment (as reconstructed for the broader area or interval within the geological formation) as explanatory factors (Fig. 3). Broad overlap exists in sites preserved as crevasse splays or in-channel deposits, with these two representing the depositional setting of the vast majority of sites (Fig. 3a, red and green polygons), though sites preserved as shoreface deposits did cluster separately from other sites (Fig. 3a, blue polygon). Palaeogeographic sampling location was effective at separating site clusters in certain situations, such as for sites in the time-equivalent portion of the upper Oldman and pre-LCZ Dinosaur Park formations (Fig. 3b, dark red and blue polygons). When expanded to all sites, sampling location did not produce distinct clusters, and broad overlap was found relating to the position of lower Belly River Group sites from Milk River and upper Belly River Group sites from DPP (Fig. 3b, light red and blue polygons). When the stratigraphic interval of each site was analyzed as a clustering variable, considerable overlap was found and no directional organization could be found that would be consistent with a linear relationship through time (Fig. 3c). Sites from the lower and middle (Comrey sandstone) Oldman Formation (Fig. 3c, purple and yellow polygons) plotted adjacent to one another, with a broad overlapping distribution of sites from the upper Oldman and pre-LCZ Dinosaur Park formations (Fig. 3c, green polygon). Most Foremost Formation sites (Fig. 3c, blue polygon) plotted between pre-LCZ Dinosaur Park Formation sites from high in stratigraphic section (Fig. 3c, sites of green polygon with more negative positions on first RDA axis), near the boundary with the LCZ, and sites from within the Lethbridge Coal Zone itself (Fig. 3c, light blue polygon). The exception to this was the ‘SPS’ site, which plotted most closely to lower Oldman Formation sites. When using palaeoenvironment as a factor, sites were assigned to one of four settings, based on their lithology and predominant fauna: (1) marine, (2) transitional, (3) terrestrial (paralic; lower coastal plain), and (4) terrestrial (alluvial; upper coastal plain). The two terrestrial groupings correspond to the palaeoenvironmental conditions from which the vast majority of dinosaur fossils are known, and represent the two primary environmental regimes discussed in previous studies of dinosaur environmental sensitivity and/or provinciality/endemism [1–7, 9, 19, 20, 22–25, 36, 37, 42–45, 49, 70]. Three non-overlapping grouping were obtained: one for sites with palaeoenvironments reconstructed as marine (Fig. 3d, blue polygon), one for sites reconstructed as transitional and preserving a mix of marine and terrestrial sedimentological features and taxa (Fig. 3d, purple polygon), and a final grouping for sites reconstructed as being terrestrial (Fig. 3d, green and yellow polygons). Sites with terrestrial palaeoenvironments were further subdivided based on their prior associations with more paralic, lower coastal plains (Fig. 3d, green polygon) or more alluvial, upper coastal plains (Fig. 3d, yellow polygon). These further subdivisions followed a clustering pattern consistent with other sites, with more inland terrestrial sites plotting further from marine and transitional sites than more coastal plain terrestrial sites. Despite this trend, considerable overlap exists between more coastally influenced terrestrial sites and more alluvial terrestrial sites, indicating that the two cannot be considered truly distinct for the purpose of site clustering.

### Pair-wise site assemblage similarity

**Fig. 4:**
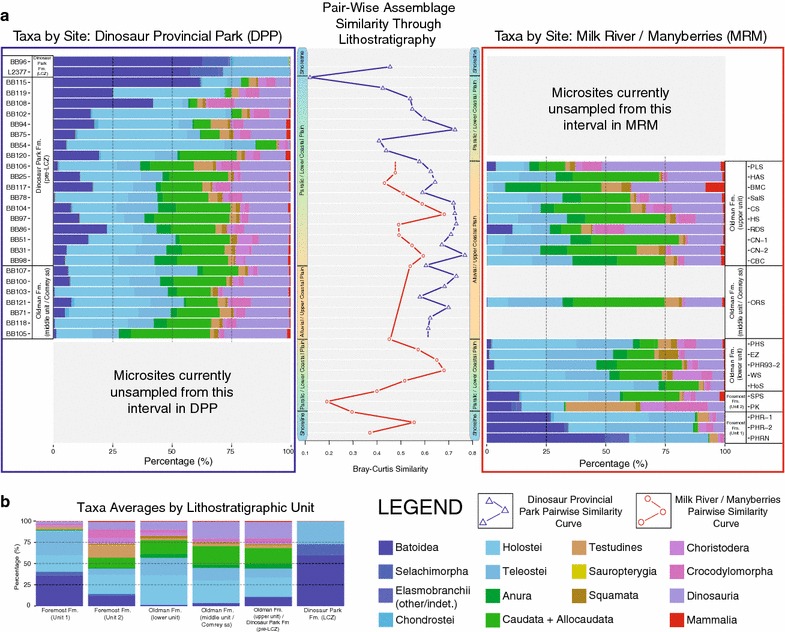
**a** Pair-wise Bray-Curtis similarity of Dinosaur Provincial Park (*blue square*) and Milk River/Manyberries (*red square*) vertebrate microfossil relative abundance assemblages through Belly River Group lithostratigraphic record. **b** Formational average proportions of each taxonomic group in sampled regions Taxonomic groups, site identifications, and other relevant information noted in legend

Pair-wise Bray-Curtis (percentage-difference) similarity was computed for each consecutive pair of stratigraphically-ordered neighbouring sites from each sampling region (DPP and MRM), along with a visual representation of relative taxonomic group abundance at each site (Fig. 4). The Milk River/Manyberries sites (Fig. 4, red box at right) range stratigraphically from the Foremost Formation to the upper unit of the Oldman Formation (time-equivalent to the pre-LCZ Dinosaur Park Formation in DPP), and the Dinosaur Provincial Park sites (Fig. 4, blue box at left) stratigraphically range from the middle Oldman Formation (‘Comrey sandstone’) to the Lethbridge Coal Zone. Pair-wise similarity curves are relatively stable for much of the sampled intervals in both DPP (Fig. 4, blue curve) and MRM (Fig. 4, red curve), ranging from approximately 50–80% similarity. Two exceptions to this stability exist, one during the regressive phase recorded near the end of the Foremost Formation in MRM sites (Fig. 4, near base of red curve) and the other during the transgressive phase in the Lethbridge Coal Zone near the boundary between the Belly River Group and the overlying Bearpaw Formation in DPP sites (Fig. 4, near top of blue curve). In both of these cases, site similarity dropped to approximately 10–20%. Trends recorded across relative abundances of taxa in individual sites (Fig. 4, DPP sites in blue box at left and MRM sites in red box at right) and in formational average taxon abundance (Fig. 4, top right) both show that the site similarity drop in the Foremost Formation was associated with large reductions in relative abundances of chondrichthyans and large increases in relative abundances of lissamphibians, with the inverse seen in the similarity drop in the Lethbridge Coal Zone.

### Sub-sample analyses of time-equivalent sites

**Fig. 5:**
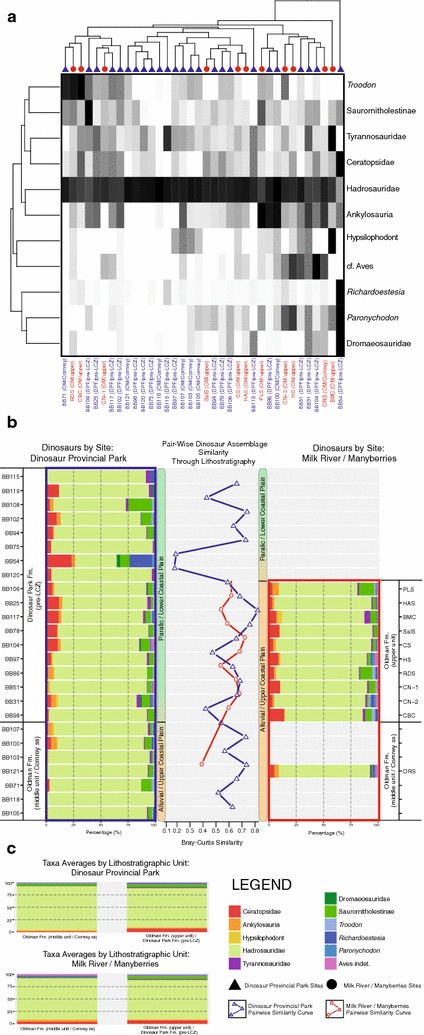
**a** R-mode vs. Q-mode cluster analysis of dinosaur component of Belly River Group microsites from the time-equivalent interval of the Oldman and Dinosaur Park formations of Dinosaur Provincial Park and Milk River/Manyberries. Dinosaur Provincial Park sites indicated with *blue text* and *triangles*. Milk River/Manyberries sites indicated with *red text* and *circles*. **b** Pair-wise Bray-Curtis similarity of Dinosaur Provincial Park (*blue square*) and Milk River/Manyberries (*red square*) microsite dinosaur relative abundance assemblages through lithostratigraphic record of time-equivalent Oldman and Dinosaur Park formations. **c** Formational average proportions of each dinosaur group in sampled regions. Taxonomic groups, site identifications, and other relevant information noted in legend

**Fig. 6:**
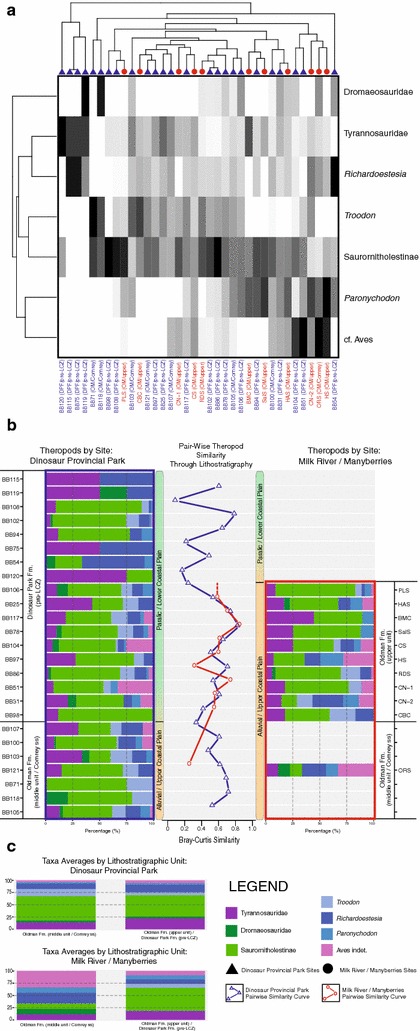
**a** R-mode vs. Q-mode cluster analysis of theropod component of Belly River Group microsites from the time-equivalent interval of the Oldman and Dinosaur Park formations of Dinosaur Provincial Park and Milk River/Manyberries. Dinosaur Provincial Park sites indicated with *blue text* and *triangles*. Milk River/Manyberries sites indicated with *red text* and *circles*. **b** Pair-wise Bray-Curtis similarity of Dinosaur Provincial Park (*blue square*) and Milk River/Manyberries (*red square*) microsite theropod relative abundance assemblages through lithostratigraphic record of time-equivalent Oldman and Dinosaur Park formations. **c** Formational average proportions of each theropod group in sampled regions. Taxonomic groups, site identifications, and other relevant information noted in legend

**Fig. 7:**
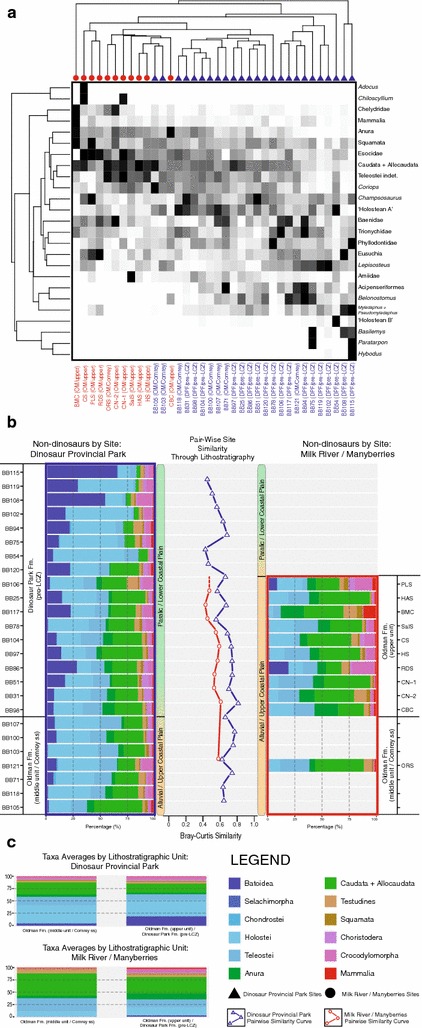
**a** R-mode vs. Q-mode cluster analysis of non-dinosaur component of Belly River Group microsites from the time-equivalent interval of the Oldman and Dinosaur Park formations of Dinosaur Provincial Park and Milk River/Manyberries. Dinosaur Provincial Park sites indicated with *blue text* and *triangles*. Milk River/Manyberries sites indicated with *red text* and *circles*. **b** Pair-wise Bray-Curtis similarity of Dinosaur Provincial Park (*blue square*) and Milk River/Manyberries (*red square*) microsite non-dinosaurian vertebrate relative abundance assemblages through lithostratigraphic record of time-equivalent Oldman and Dinosaur Park formations. **c** Formational average proportions of each non-dinosaurian vertebrate group in sampled regions. Taxonomic groups, site identifications, and other relevant information noted in legend

Using three subsets of the broader relative abundance dataset, with re-sampled values of dinosaurs from Brinkman et al. [8] in place of Brinkman [12] for the first two, R- vs. Q-mode cluster analyses and pair-wise assemblage similarity analyses were performed for dinosaur-only (Fig. 5), theropod-only (Fig. 6), and non-dinosaur (Fig. 7) components of the assemblage. These subset comparisons were made only for sites in the overlapping stratigraphic intervals of each sampling area, namely the middle (‘Comrey sandstone’) Oldman Formation, and the upper Oldman Formation and pre-LCZ Dinosaur Park Formation.

In the dinosaur sub-sample (Fig. 5), hadrosaurs constituted the vast majority of assemblage relative abundance in almost all sites from both Dinosaur Provincial Park and Milk River. The only exception to this was the DPP ‘BB54’ site, which clustered away from all other sites (Fig. 5A). With the exception of hadrosaurs, no single dinosaur taxon was found to be driving large-scale site clustering, though increased *Troodon* relative abundance seems to drive the finer-scale clustering of two MRM sites (‘RDS’ and ‘CBC’) and a DPP site (‘BB71’), and relative abundance of ankylosaurs drives the clustering of two DPP sites (‘BB86’ and ‘BB100’) and one MRM site (‘PLS’). Unlike in the cluster analyses of the broader vertebrate assemblages, there is less distinct clustering of sites by sampling region (Fig. 5a, MRM sites indicated by red circles and DPP sites indicated by blue triangles). Pair-wise site similarity for dinosaur assemblages in the sub-sampled interval is relatively stable for both DPP and MRM, ranging approximately 40–80% similarity (Fig. 5b, DPP sites in blue box and represented by blue similarity curve and MRM sites in red box and represented by red similarity curve). The only deviation from this trend is a drop to approximately 20% similarity for sites neighbouring ‘BB54’, which as noted above represents an apparent outlier due to a lower relative abundance of hadrosaurs, and much higher relative abundances of ceratopsians and *Richardoestesia* (Fig. 5b). Formational average abundances of dinosaurs (Fig. 5c) do not show any major shifts in assemblage between the middle Oldman Formation sites and the sites of the time-equivalent upper Oldman and pre-LCZ Dinosaur Park formations, nor is there considerable difference in assemblages between the DPP and Milk River localities. The only exceptions to this are a moderate increase in ceratopsians in DPP between the middle Oldman Formation (~1% relative abundance) and time-equivalent pre-LCZ Dinosaur Park Formation (~7% relative abundance), a shift also found in the equivalent intervals of the Milk River sites (~4 to ~7% relative abundance, respectively). In the middle Oldman Formation and time-equivalent upper Oldman Formation of Milk River, there were also changes in small theropod relative abundances, with saurornitholestines increasing slightly (~1 to ~5%), and *Troodon* going from absent to present (with relative abundance in the upper Oldman Formation of ~1%).

The theropod-only sub-sample analyses (Fig. 6) produced similar results to the sub-sample of dinosaurs. Most sites did not show any strong signal from a particular taxon driving clustering patterns (Fig. 6a), though a few clustered together due to their greater association with tyrannosaurids and *Richardoestesia* (‘BB120’, ‘BB115’, ‘BB75’, BB119) or with cf. *Aves* (‘BB61’, ‘CN-2’, ‘ORS’, ‘HS’). As with dinosaurs, the site similarity curves of the theropod sub-samples from DPP and MRM are very similar (Fig. 6b), both staying within a range of ~30 to ~80% similarity. The lower bound of that similarity range related to sites neighbouring those with very little theropod material (e.g. ‘BB120’, ‘BB75’, ‘BB115’, and ‘BB119’). The formational average theropod relative abundances (Fig. 6c) in DPP show no appreciable differences, with slight increases in proportions of tyrannosaurids and *Richardoestesia*, and slight decrease in *Troodon*. In MRM, there are more considerable differences in formational average relative theropod abundances, with saurornitholestine proportions greatly increasing, *Troodon* appearing in the upper Oldman Formation while not being found in the middle Oldman Formation, and all other taxa proportionally decreasing slightly.

The non-dinosaur sub-sample analysis (Fig. 7) produced similar results to the non-marine components of the R- vs. Q-mode analysis of all microsite data (Fig. 2, yellow square). Clustering of sites based on their provenience was apparent, though sites did not cluster exclusively based on being from DPP or MRM (Fig. 7a). Sites from MRM clustered more closely to the majority of DPP sites than a number of DPP sites (e.g. ‘BB106’, ‘BB117’, ‘BB121’, ‘BB94’, ‘BB75’, ‘BB119’, ‘BB102’), with those latter sites forming a cluster more similar to each other than to any other site. This cluster was associated the proportion of particular actinopterygians (e.g. *Lepisosteus*, *Paratarpon*, Acipenseriformes, *Belonostomus*), and turtles (e.g. *Basilemys*, Baenidae, Trionychidae). Two sites, both stratigraphically high in the DPF and close to the LCZ, were associated with batoids (e.g. *Myledaphus* + *Pseudomyledaphus*), *Basilemys*, and in one case sharks (*Hybodus*, in ‘BB115’). As in other analyses, ‘BB54’ grouped as something of an outlier, and was here associated strongly with ‘Holostean A’ and ‘Holostean B’. Other DPP sites were broadly associated with many taxa, though in particular with actinopterygians (e.g. *Coriops*, ‘Holostean A’), baenid turtles, and lissamphibians (e.g. Caudata + Allocaudata). Sites sampled from MRM as a whole were distinguished from DPP mainly due to an even stronger association with lissamphibians (e.g. Caudata + Allocaudata) and certain actinopterygians (e.g. esocids, teleosts), though particular MRM sites were also distinguished based on their association with taxa that were either absent or in low abundance at other sites, particularly those from DPP. For example, *Adocus* and *Chiloscyllium* are strongly associated with the ‘CS’ site, though absent or in very low abundance in most sites. One MRM site (‘BMC’) clustered as an outlier, and was distinguished through a suite of taxa (e.g. chelydrid turtles, mammals, anurans, squamates). Both of these outlier sites (‘BB54’ and ‘BMC’) have been noted in previous research to be sedimentologically distinct from other sites, possibly representing an exception to the general trend of depositional setting having a relatively small effect on microsite assemblage structure [14, 52]. Site similarity curves for DPP and MRM (Fig. 7b) were very similar, and very stable, both fluctuating around 60% similarity for much of the sampled interval. The only prominent exception to this came at the top of the time-equivalent interval in DPP, where similarity began to steadily drop, reaching approximately 40% similarity by the top of the sampled interval. Overall proportions of major taxonomic groups in DPP and MRM during this interval (Fig. 7c) were similar, with the notable exception being the higher proportion of batoids in DPP (<20%) when compared to MRM (<5%).

## Discussion

### Drivers of faunal assemblage clustering in the Belly River Group

Our results broadly support the conclusions of previous studies such as Brinkman et al. [14], expand on their work to include sites from both MRM and DPP in a series of analyses, and more thoroughly test the role that various abiotic factors play in structuring the preserved faunal assemblages. The results of the R- vs. Q-mode cluster analyses (Fig. 2), RDA analyses (Fig. 3), and pair-wise site similarity comparisons (Fig. 4) for the full sample of microsite faunal assemblages of MRM and DPP indicate that palaeoenvironmental changes, particularly marine-terrestrial transitions, are responsible for the most significant changes in faunal assemblage structure, and that other factors like site depositional setting, relative stratigraphic position within the Belly River Group, and palaeogeographic sampling location played a lesser to negligible role. While depositional setting (Fig. 3a) appears superficially to explain site clustering, it should be noted that the only depositional setting that did not display considerable overlap with others were shoreface deposits, and each site characterized as a shoreface deposit was also characterized as preserving a primarily marine palaeoenvironment. Of the three depositional settings analyzed there was broad overlap in sites characterized as crevasse splays or in-channel deposits (Fig. 3a), which is consistent with palaeoenvironment (Fig. 3d) driving site clustering, as there is also considerable overlap in terrestrial sites, all of which in this sample are preserved as either in-channel or crevasse splay deposits. The differing depositional characteristics of these terrestrial sites are therefore not having a strong effect on the preserved faunal assemblage. Palaeogeographic sampling location appears to also have some effect on faunal assemblage structure, at least within sites from the time-equivalent interval of the Oldman and pre-LCZ Dinosaur Park formations of Dinosaur Provincial Park and Milk River (Fig. 3b). In that context, the DPP and MRM sites formed distinct clusters, though this separation did not hold when expanded to the complete sample of Belly River Group microsites. The separate time-equivalent DPP and MRM clusters provide further support to the hypothesis that at least some of the differences in microsite faunal assemblage structure is the result of endemism related to environmental variation across the palaeolandscape [12, 14]. However, the distinct clustering of these sites may also be partially related to the absence of several taxa (e.g. *Paronychodon*, cf. Aves, *Richardoestesia*) in the DPP microsite data that are moderately abundant in MRM sites, despite these taxa being reported in the Dinosaur Park Formation [71]. This effect is similarly seen in the PHRN site (Fig. 2) being associated with ‘Theropoda indet’ material (alongside the numerous marine chondrichthyan taxa that primarily characterize the site) due to this taxonomic category only existing for this site (based on the source data).

Overall, the results of these analyses for the entire Belly River Group microsite database build on prior research conducted on this subject [8, 12–14, 16, 53, 58, 61], and serve to more thoroughly and quantitatively establish the patterns that have been observed in this system. It is not particularly surprising that changes in sea level in the Belly River Group acted as a strong driver of environmental and faunal assemblage change, as the most significant change in faunal assemblage during these intervals is the inverse proportional change in chondrichthyans and lissamphibians, which is almost certainly related to the degree of marine preference (or lack-thereof in the latter case) in these taxa [16, 72, 73]. However, it is important to quantify and understand the exact nature of these trends, as these data form the baseline for future comparisons and facilitate the testing of more hotly-debated questions, such as the environmental sensitivity of dinosaurs and the effects that more subtle environmental variation have on local palaeocommunity structure.

### Altitudinal and latitudinal sensitivity of dinosaur assemblages

The sensitivity of dinosaur populations to changes in altitudinal (distance from palaeoshoreline) and latitudinal environmental gradients has been the subject of considerable debate for over 30 years [1–9, 11, 15, 23, 26, 27, 42–46, 55, 62, 70], and though it has been questioned [17, 18, 22, 28, 74], it remains one of the primary explanations for patterns observed in the evolution and distribution of dinosaurs throughout the Late Cretaceous of western North America. A focused sub-sampling of the time-equivalent interval of the Oldman and Dinosaur Park formations within the larger Belly River Group microsite abundance dataset facilitates a controlled and direct test of dinosaur assemblage changes across differing palaeoenvironments (Figs. 5, 6), while also allowing comparisons to the non-dinosaur component of the broader vertebrate assemblage (Fig. 7).

Within dinosaurs (Fig. 5) there is broad assemblage stability, despite the sampled regions representing differing terrestrial environments (lower coastal plain vs. upper coastal plain/inland alluvial fan). In both DPP and MRM, hadrosaurs dominate the preserved dinosaur assemblages, often representing over 80% of relative abundance. A moderate increase in ceratopsians is noted through time in DPP, though across time-equivalent intervals the proportional difference between ceratopsians in DPP and MRM is negligible. This stability between sampling areas is also generally seen in the theropods (Fig. 6), with *Troodon* representing the main exception, as it is not present in the earliest MRM site, appearing there later than in DPP. This was also noted by Brinkman et al. [14], and was attributed to migration/range-expansion. The later appearance of *Troodon* in MRM, along with an increase in saurornitholestine material from the middle to upper Oldman Formation in MRM, may be a genuine change in theropod assemblage that is not seen in DPP, or it may be a result of sampling issues, as the middle Oldman Formation of the MRM area is only represented by a single site (‘ORS’). The non-dinosaur component of the vertebrate faunal assemblage was relatively stable across much of the sampled interval, although there were considerable differences in the relative abundances of taxa between DPP and MRM (Fig. 7), with batoids forming a much larger component of the overall fauna in DPP. Unlike the pattern shown in the broader vertebrate assemblages (Fig. 7), there does not appear to be strong clustering according to sampling region in dinosaurs (Figs. 5a, 6a), suggesting that the palaeogeographic signal in the broader analysis is due to abundance differences (e.g. batoids) or rare/endemic taxa of non-dinosaurian affiliation (e.g. lissamphibians, turtles, etc.). The similarity of faunal assemblages, and particularly dinosaur assemblages, within the two terrestrial palaeoenvironmental settings (coastal plain vs. alluvial/inland) indicates that these subtle variations in terrestrial palaeoenvironment may have less effect on faunal assemblage structure than previously suggested [1, 12, 14], a position supported by recent research on ceratopsids in the Oldman Formation of the Milk River/Manyberries region [22]. It is also possible that the palaeoenvironmental interpretation of these formations and sampling areas is more complex than originally described [50], though, pending future geological revisions, there is currently no reason to think this is the case. The relative similarity of the dinosaur faunal assemblages of DPP and MRM, and how those contrast to the differences seen in the rest of the vertebrate faunal assemblage between these areas and throughout the extent of the Belly River Group, runs counter to the long-standing idea that dinosaurs, including large bodied taxa like hadrosaurs and ceratopsians, are sensitive to relatively small environmental changes across the palaeolandscape, and that this sensitivity is the cause of the large diversity of geographically or formationally restricted taxa known from the Late Cretaceous of western North America [1–8, 11, 19–24, 27, 39, 43, 70].

## Conclusions

The results of this study demonstrate that palaeoenvironmental setting is the primary driver of differences in vertebrate faunal assemblages throughout the Belly River Group, with palaeogeography/palaeolandscape acting as another factor in structuring these assemblages. Depositional setting and stratigraphic interval do not have particularly strong effects on the preserved faunal assemblage, confirming the results of other recent studies [14, 59].

The sub-sample analyses of dinosaur and theropod assemblages, and their comparisons to the broader vertebrate assemblages, suggest one of two possible conclusions: either (a) dinosaurs are not sensitive to subtle changes in altitudinal and latitudinal palaeoenvironmental gradients, and/or (b) the differences in environment between the pre-LCZ Dinosaur Park Formation of DPP and the upper Oldman Formation of MRM have been overstated. The higher proportion of batoids in DPP than MRM across this same interval suggests that the more coastally-influenced terrestrial environment of DPP is genuine, providing evidence against the long-held idea that dinosaur communities were particularly sensitive to small-scale environmental gradients, such as paralic (coastal) to alluvial (inland) regimes within a single depositional basin. Further research is required to fully answer this question, though it is possible that consistently high rates of evolution and niche partitioning among species within each of the sampled dinosaur families were more responsible for the high diversity and frequent turnovers in dinosaur taxa throughout the Late Cretaceous of North America than any particular sensitivity to subtle environmental change.

## Additional files

**Additional file 1.** Taxon abundance table of dinosaurs for sites from time-equivalent Oldman and Dinosaur Park formations. Milk River/Manyberries source data consistent with Table 1, while Dinosaur Provincial Park data derived from targeted dinosaur re-sampling of Brinkman et al [8].

**Additional file 2.** Environmental factors associated with each Belly River Group microfossil site, for use in redundancy analysis.

## Abbreviations

BRG: Belly River Group
DPF: Dinosaur Park Formation
DPP: Dinosaur Provincial Park
LCZ: Lethbridge Coal Zone
MRM: Milk River/Manyberries
OM: Oldman Formation
RDA: redundancy analysis
UPGMA: unweighted pair group method with arithmetic mean
