# Erratum to: Palaeoenvironmental drivers of vertebrate community composition in the Belly River Group (Campanian) of Alberta, Canada, with implications for dinosaur biogeography

**DOI:** 10.1186/s12898-016-0111-y

**Published:** 2017-01-09

**Authors:** Thomas M. Cullen, David C. Evans

**Affiliations:** 1 Department of Ecology and Evolutionary Biology, University of Toronto, Toronto, ON Canada; 2 Department of Natural History, Royal Ontario Museum, 100 Queen’s Park, Toronto, ON M5S 2C6 Canada

## Erratum to: BMC Ecol (2016) 16:52 DOI 10.1186/s12898-016-0106-8

After publication of the original article [1], it was brought to our attention that the phrase ‘a lack of sensitivity to subtle environmental gradients casts doubt on these forces acting as a driver of putative endemism of dinosaur populations in the Late Cretaceous of North America’ in “Conclusion” section should read ‘a lack of sensitivity to subtle environmental gradients casts doubt on these forces acting as a driver of altitudinal zonation of dinosaur communities in the Late Cretaceous of North America’.

The phrase ‘this hypothesis is based on the resilience to environmental variation and broad latitudinal distributions seen in many groups of large mammals today [2, 7, 32], though it is at odds with much of the literature on dinosaur environmental associations [1, 2, 3, 4, 5, 6, 7, 8, 9, 11, 14, 15, 19, 20, 21, 22, 23, 24, 26, 27, 42, 43, 62]’ in “Background” section should read ‘although it is frequently hypothesized that large dinosaurs are sensitive to environmental variation [1, 2, 3, 4, 5, 6, 7, 8, 9, 11, 14, 15, 19, 20, 21, 22, 23, 24, 26, 27, 42, 43, 62], many groups of large mammals today are resilient to environmental variation and have broad latitudinal distributions [2, 7, 32]’.

The phrase ‘the ability to test hypotheses about dinosaur biogeography, endemism, and environmental sensitivity has historically been difficult, as many species were collected with only limited geological data or stratigraphic information, and were known by very low sample sizes [24, 43, 47], though ongoing work relocating these sites and incorporating them into the broader stratigraphy is ameliorating some of these issues [23, 24, 43, 44, 48]’ in “Background” section should read ‘the ability to test hypotheses about dinosaur biogeography, endemism, and environmental sensitivity has historically been difficult, as many specimens were collected with only limited geological data or stratigraphic information, and known by very low sample sizes [24, 43, 47], though ongoing work collecting new specimens, relocating these sites, and incorporating them into the broader stratigraphy is ameliorating some of these issues [23, 24, 43, 44, 48]’.

The phrase ‘the full extent of the Belly River Group records two major regional sea level changes in the Western Interior Seaway (the relatively shallow, inland seaway that at its greatest extent stretched from The Arctic Ocean to Gulf of Mexico), the first of which is a regressive event in the Foremost and lower Oldman formations, and the second of which is a major transgressive event recorded in the uppermost Oldman and Dinosaur Park formations that marks the boundary between the Belly River Group and the overlying Bearpaw Formation [49, 50]. The Foremost Formation is the stratigraphically lowest unit within the Belly River Group, and gradationally overlies the marine shales of the Pakowki Formation’ in “Background” section should read ‘the full extent of the Belly River Group records two major regional sea level changes in the Western Interior Seaway (the relatively shallow, inland seaway that at its greatest extent stretched from the Arctic Ocean to the Gulf of Mexico), the first of which is a regressive event in the Foremost and lower Oldman formations, and the second of which is a major transgressive event recorded in the uppermost Oldman and Dinosaur Park formations that marks the transition between the Belly River Group and the overlying Bearpaw Formation [49, 50]. The Foremost Formation is the stratigraphically lowest unit within the Belly River Group, and conformably overlies the marine shales of the Pakowki Formation’.

The phrase ‘given the ongoing debate regarding the putatively narrow associations of dinosaurs with particular environments, locations, and/or geological formations, this study seeks to use the largest Cretaceous vertebrate microsite dataset yet assembled to first confirm the previously suggested associations between faunal assemblages and differing environments, and then use those as a proxy to test for differences in dinosaur assemblages in the time-equivalent sections of the Dinosaur Park and Oldman formations’ in “Background” section should read ‘given the ongoing debate regarding the putatively narrow associations of dinosaurs with particular environments, locations, and/or geological formations, this study uses the largest Cretaceous vertebrate microsite dataset yet assembled to first test the previously suggested associations between faunal assemblages and differing environments, and then use those as a proxy to test for differences in dinosaur assemblages in the time-equivalent sections of the Dinosaur Park and Oldman formations’.

The phrase ‘the separate time-equivalent DPP and MRM clusters provide further support to the hypothesis that at least some of the differences in microsite faunal assemblage structure is the result of endemism related to environmental variation across the palaeolandscape’ in “Discussion” section should read ‘the separate time-equivalent DPP and MRM clusters provide further support to the hypothesis that at least some of the differences in microsite faunal assemblage structure is the result of biogeographic differences related to environmental variation across the palaeolandscape’.

In addition, the phrase ‘it is also possible that the palaeoenvironmental interpretation of these formations and sampling areas is more complex than originally described [50], though, pending future geological revisions, there is currently no reason to think this is the case. The relative similarity of the dinosaur faunal assemblages of DPP and MRM, and how those contrast to the differences seen in the rest of the vertebrate faunal assemblage between these areas and throughout the extent of the Belly River Group, runs counter to the longstanding idea that dinosaurs, including large bodied taxa like hadrosaurs and ceratopsians, are sensitive to relatively small environmental changes across the palaeolandscape, and that this sensitivity is the cause of the large diversity of geographically or formationally restricted taxa known from the Late Cretaceous of western North America [1, 2, 3, 4, 5, 6, 7, 8, 11, 19, 20, 21, 22, 23, 24, 27, 39, 43, 70]’ in “Discussion” section should read ‘it is also possible that the palaeoenvironmental interpretation of these formations and sampling areas is more dynamic than originally described [50], though, pending future geological investigations, there is currently no reason to think this is the case. The relative similarity of the dinosaur faunal assemblages of DPP and MRM, and how those contrast to the differences seen in the rest of the vertebrate faunal assemblage between these areas and throughout the extent of the Belly River Group, does not support the idea that dinosaurs, including large bodied taxa like hadrosaurs and ceratopsians, are sensitive to relatively small environmental changes across the regional palaeoenvironmental landscape. How this plays out over continental scales and between basins is currently unclear, but at least in terms of community composition measured at the family level, large bodied herbivore communities seem to exhibit little variation over the altitudinal transects considered here [1, 2, 3, 4, 5, 6, 7, 8, 11, 19, 20, 21, 22, 23, 24, 27, 39, 43, 70]’.

The phrase ‘the subsample analyses of dinosaur and theropod assemblages, and their comparisons to the broader vertebrate assemblages, suggest one of two possible conclusions: either (a) dinosaurs are not sensitive to subtle changes in altitudinal and latitudinal palaeoenvironmental gradients, and/or (b) the differences in environment between the pre-LCZ Dinosaur Park Formation of DPP and the upper Oldman Formation of MRM have been overstated. The higher proportion of batoids in DPP than MRM across this same interval suggests that the more coastally-influenced terrestrial environment of DPP is genuine, providing evidence against the long-held idea that dinosaur communities were particularly sensitive to small-scale environmental gradients, such as paralic (coastal) to alluvial (inland) regimes within a single depositional basin. Further research is required to fully answer this question, though it is possible that consistently high rates of evolution and niche partitioning among species within each of the sampled dinosaur families were more responsible for the high diversity and frequent turnovers in dinosaur taxa throughout the Late Cretaceous of North America than any particular sensitivity to subtle environmental change’ in “Conclusion” section should read ‘the subsample analyses of dinosaur and theropod assemblages, and their comparisons to the broader vertebrate assemblages, suggest one of two possible conclusions: either (a) dinosaur community composition is not sensitive to subtle changes in altitudinal and latitudinal palaeoenvironmental gradients, and/or (b) the differences in environment between the pre-LCZ Dinosaur Park Formation of DPP and the upper Oldman Formation of MRM have been overstated. The higher proportion of batoids in DPP than MRM across this same interval suggests that the more coastally-influenced terrestrial environment of DPP is genuine, refuting the long-held idea that dinosaur communities were particularly sensitive to small-scale environmental gradients, such as paralic (coastal) to alluvial (inland) regimes within a single depositional basin. Further research is required to fully answer this question, but it is possible that consistently high rates of evolution and niche partitioning among species within each of the sampled dinosaur families played a greater role in the observed high diversity and frequent turnovers in dinosaur taxa throughout the Late Cretaceous of North America’.

Furthermore, the phrase ‘Derek Larson, Karma Nanglu, Kentaro Chiba, Caleb Brown, Kirstin Brink, and Nicolas Campione are thanked for additional discussions related to this research’ should read ‘Michael Ryan, Derek Larson, Karma Nanglu, Kentaro Chiba, Caleb Brown, Kirstin Brink, Jordan Mallon, and Nicolas Campione are thanked for additional discussions related to this research’.

The original article was corrected. The updated phrases have been published in this erratum for quick reference. We apologise for any confusion this may have caused.
